# Mental Health of Nepalese Migrant Workers: A Call for Action in South Korea

**DOI:** 10.31729/jnma.9165

**Published:** 2025-07-31

**Authors:** Bibek Giri, Minani Gurung, Qorinah Estiningtyas Sakilah Adnani, Vijay Kumar Chattu

**Affiliations:** 1 Research Institute for Collaborative Development, Kathmandu, Nepal; 2 School of Medical Sciences, Kathmandu University, Nepal; 3 Department of Public Health, Faculty of Medicine, Padjadjaran University, Bandung, Indonesia; 4 Department of Occupational Science & Occupational Therapy, Temerty Faculty of Medicine, University of Toronto, Canada; 5 Center for Global Health Research, Saveetha Medical College and Hospital, Saveetha Institute of Medical and Technical Sciences (SIMATS), Saveetha University, Chennai, India

**Keywords:** *COVID-19*, *health diplomacy*, *mental health*, *Nepalese migrant workers*

## Abstract

Mental health problems among migrants is a serious issue around the globe. Nepalese migrant workers in South Korea are facing serious mental health problem that affects not only the people involved but also the society at large. Moreover, the COVID-19 pandemic has worsened the already dire mental health situation of Nepali workers. Global health diplomacy can be a key factor in addressing mental health by engaging actors from various domains to evaluate mental health in global health priorities. This article reviews the current state of mental health and discusses the recent development in mental health among Nepalese migrant workers in South Korea.

## INTRODUCTION

South Korea has emerged as one of the most appealing destinations for Nepalese migrant workers since the introduction of the Employment Permit System (EPS). As of now, there are around 42,000 Nepalis residing in South Korea, mainly as migrant workers highlights both opportunities and significant challenges, specially concerning mental health.^1^ Despite economic benefits, workers face stressors such as discrimination, isolation and despair, necessitating urgent attention.^2^ This viewpoint underscores the need for targeted interventions and policy measures to mitigate mental health risks, ensuring safer and more supportive environment for migrant’s workers.

## FOCUS ON MENTAL HEALTH CRISIS

The world is witnessing a massive movement of people across borders, with one out of every eight people on the planet being migrant.^3^ In 2020, the world had about 281 million people who lived outside their countries of origin, representing 3.6 per cent of all humans on the planet.^4^ This is the latest global estimate of international migration. These migrants depart from their homeland in pursuit of work prospects, often encountering various obstacles and difficulties in their journey. They are motivated by a need to sustain themselves and their loved ones, but the reality is quite different from what they had envisioned.

A survey carried out in South Korea revealed that the rate of mental health problems among Nepalese migrants was 23%.^5^ Despite these worrisome figures, the mental well-being of Nepalese migrants in South Korea is frequently ignored and disregarded. It is vital to comprehend the specific situations and difficulties encountered by Nepalese migrants in South Korea.^5^ Research conducted in Korea’s Gyeonggi province revealed that discrimination was a prevalent issue among migrant workers. More than 66% of them reported facing some form of unfair treatment.^6^ The mental health crisis among Nepalese migrants in South Korea is alarming, as evidenced by the high mortality rate, with many cases of suicide. Recent research has shown the seriousness of mental health issues among Nepalese migrant workers in South Korea, with a large number of deaths being linked to suicide. Atteray et al. reported that 47 out of 170 deaths among Nepalese migrant workers in South Korea were by suicide.^2,6^ This is an indicator for both the origin and destination countries to take decisive steps to meet the mental health requirements of these at-risk workers.

Living and working in South Korea as a Nepalese migrant is not an easy feat. It entails overcoming various obstacles that can take a toll on one’s psychological and emotional state. From the language barrier and adapting to a different culture to facing unfair treatment, exhausting labour, harsh environments, loneliness, and inadequate assistance, these are some of the hardships that Nepalese migrants endure in South Korea.^7^ The well-being of Nepalese migrants in South Korea is not only a personal matter but also a public concern. Their mental health issues, if neglected, can jeopardize their safety and quality of life, as well as affect their work performance, health expenditure, and social relationships.^5^ Furthermore, unresolved mental health problems can have lasting adverse consequences on individuals and their families. Therefore, it is vital to recognize the role of mental health in shaping the overall health and productivity of Nepalese migrants. Moreover, addressing their mental health needs can foster a more welcoming and compassionate society that honors the rights and dignity of all people.

## MENTAL HEALTH EMERGENCIES IN NEPALESE WORKERS

Nepalese workers in South Korea are facing a severe mental health problem that requires urgent intervention. Survey data shows that 23% of Nepalese migrants in South Korea suffer from mental health issues, which is similar to other migrant populations who encounter various difficulties.^5^ This disturbing figure underscores the necessity of taking measures to enhance the mental health of Nepalese migrant workers. Nepalese migrant workers in South Korea frequently face barriers to accessing psychosocial support services because of their lack of residency rights.^8^ In addition, The COVID-19 pandemic has worsened the already dire mental health situation of Nepalese migrant workers in South Korea. The pandemic has imposed more difficulties, such as unstable employment, economic hardship, and social detachment. These difficulties, along with the existing disadvantages of migrant workers, have profoundly affected their mental health. The high mortality and suicide rates among Nepalese migrant workers in South Korea demand immediate intervention to safeguard their mental well-being.^6^ The mental health problem of Nepalese migrant workers in South Korea is a serious issue that affects not only the people involved but also the society at large. Individuals who are dealing with mental health issues may experience symptoms such as anxiety, depression, stress, and insomnia.^9^ These symptoms can hinder their ability to perform well at work and in their personal lives. This can result in reduced productivity, increased absenteeism, and even loss of employment. Furthermore, the effect of mental health issues goes beyond the individual to their families and communities. Families of migrant workers may also suffer from psychological distress and emotional strain as they see their loved ones coping with mental health issues.^9^ Communities, especially the Nepalese community in South Korea, may also be impacted as they observe the mental health problems among their fellow migrants.

## FACTORS AFFECTING MENTAL HEALTH

Nepalese migrant workers in South Korea encounter various obstacles that affect their mental health. These obstacles include communication difficulties, cultural clashes, unfair treatment, exhausting labor, harsh environments, loneliness, and insufficient assistance.^10^ Research has indicated that these obstacles, coupled with exposure to discrimination, have a significant influence on the mental health and well-being ofmigrant workers.^11^ Moreover, various studies have indicated that the Nepalese migrant worker community faces multiple risk factors for deteriorating mental health, such as the challenges of settling in, the absence of social support, the burden of financial stress, the demands of education, and the threat of unemployment.^12^ Communication problems are a major obstacle for Nepalese migrant workers in South Korea. Without being proficient in the language, these workers may find it hard to convey their needs and concerns, leading to feelings of frustration, isolation, and helplessness.^13^ Additionally, the cultural differences between Nepal and South Korea can also affect their mental health.^14^ For instance, Nepalese workers may encounter challenges in adjusting to the different work cultures, social norms, and expectations in South Korea. They may feel alienated, misjudged, and unable to fully assimilate into society, which can harm their mental well-being. Moreover, discrimination against migrant workers in South Korea adds more pressure and hardship to their mental health.^6^ Research has indicated that discrimination is a common issue among migrant workers in South Korea. This discrimination can take various shapes, such as verbal harassment, unjust treatment, and isolation. The absence of social support networks and the limited availability of suitable mental health services, as well as low health literacy, impede their access to prompt healthcare and support, worsen the mental health problem of Nepalese migrant workers in South Korea as well as Insufficient awareness of mental health among Nepalese migrant workers worsens the condition.^15^

## STRATEGIES FOR ENHANCING MENTAL HEALTH SUPPORT

The mental health difficulties of migrant workers worldwide have gained more attention in recent years. These difficulties are especially common among Nepalese migrant workers in South Korea. Addressing these mental health issues and devising effective ways to assist Nepalese migrant workers in South Korea is crucial. To tackle the mental health inequalities among Nepalese migrant workers in South Korea, a holistic intervention system is essential. This intervention system should consist of screening for psychological stress and collaborating with mental health professionals, factory medical officers, general medical practitioners, and skilled community health workers to offer extensive mental health support.^16^ Additionally, the impact of social support networks on alleviating mental health problems among Nepalese migrant workers should be taken into account.^17^ These support networks can offer a sense of community and belonging, easing feelings of stress, isolation, despair, and frustration. Studies have indicated that social support networks have a crucial role in cushioning the effects of mental health troubles among migrant workers. Hence, it is vital to give priority to the development and enhancement of social support networks that are specially designed to meet the needs of Nepalese migrant workers in South Korea. Moreover, the COVID-19 pandemic has worsened the already dire mental health situation of Nepalese migrant workers in South Korea. The pandemic has imposed more difficulties, such as unstable employment, economic hardship, and social detachment.^18^ These difficulties, along with the existing disadvantages of migrant workers, have profoundly affected their mental health. To effectively deal with these difficulties, a holistic approach is required. This approach should include not only meeting the urgent mental health demands of Nepalese migrant workers but also addressing the root causes that lead to their mental health inequalities. These factors consist of insufficient awareness of mental disorders and their lasting consequences, as well as the poor educational level of many Nepalese labor migrant and nonmigrant workers.^15^ In addition, the intervention system should also aim to increase awareness and provide education about mental health among Nepalese migrant workers. This way, we can equip them with the essential knowledge and skills to identify and manage their own mental health needs.

## ROLE OF GLOBAL HEALTH DIPLOMACY

The significance of global health diplomacy for tackling various health issues, such as mental health, has been increasingly acknowledged in recent years. Global health diplomacy is the nexus between diplomacy and global health, where diplomatic means are employed to address health problems and foster collaboration on health-related matters among countries. It involves negotiations and collaborations on global policy issues that shape and influence the global environment for health and brings together a wide range of actors in areas that affect public health.^19^ Health diplomacy can also facilitate the sharing of information, expertise, and assets among Nepal and South Korea to enhance the quality of mental health care for migrant workers. Through health diplomacy, the governments can collaborate to design and execute programs that target mental health before and after departure, offer guidance and emotional assistance, and foster the mental wellness of Nepalese migrant workers.^20^ The urgent need to address the mental health issues of Nepalese migrant workers in South Korea is undeniable. Therefore, health diplomacy can play a crucial role in improving the well-being and welfare of Nepali workers in South Korea.

Mental health is one of the most neglected and stigmatized aspects of health despite its huge burden and impact on individuals and societies.^21^ By integrating mental health into the global health agenda, global health diplomacy can create pathways for increased awareness, improved policies, and enhanced international cooperation to address mental health challenges. Global health diplomacy can be a key factor in addressing mental health by engaging actors from various domains to elevate mental health in global health priorities. By using diplomatic means and dialogues, global health diplomacy can support policies that foster mental health, allocate resources for mental health services, and decrease stigma around mental health.^22,23^ Global health diplomacy can also enable the sharing of information and expertise in mental health between Nepal and South Korea, allowing them to benefit from each other's achievements and difficulties and cooperate on novel methods of mental health care. By engaging in global health diplomacy, Nepal and South Korea can collaborate to design and execute policies, programs, and initiatives that meet the mental health needs of Nepalese migrant workers.^5^ A key aspect of global health diplomacy in tackling mental health issues is advocacy and awareness raising.^24^ Through diplomatic means, countries can cooperate to increase awareness about the mental health difficulties faced by Nepalese migrant workers in South Korea. This can include organizing joint events such as conferences, workshops, and seminars to inform policymakers, healthcare professionals, and the general public about the specific mental health needs of Nepalese migrant workers. Furthermore, global health diplomacy can enable the sharing of information and expertise between Nepal and South Korea in the field of mental health. This can include joint research projects, training programs, and capacity-building initiatives to improve the mental health services available to Nepalese migrant workers in South Korea.^25^

By supporting mental health as a global concern, health diplomacy can assist in eliminating obstacles to access and guarantee that mental health services are incorporated into primary healthcare systems; by supporting the integration of mental health services in bilateral agreements, health diplomacy can guarantee that adequate resources and assistance are designated for meeting the mental health needs ofNepalese migrant workers.^26^ Furthermore, global health diplomacy can facilitate the creation of mental health clinics or specialized centers that serve the needs of Nepalese migrant workers in South Korea. These clinics can offer mental health services that respect the culture and language of Nepalese migrant workers, ensuring that they have access to quality care.^27^ Global health diplomacy can also be a key factor in addressing the social factors that affect mental health, such as poverty, inequality, and discrimination.^28^ By participating in diplomatic dialogues and negotiations, global health diplomacy can support policies that tackle these root causes and foster mental wellness. Furthermore, global health diplomacy can enable the creation of holistic mental health policies and frameworks that cater to the specific needs of Nepalese migrant workers. ^29^ This can include cooperative endeavors between the governments of Nepal and South Korea, as well as international organizations and stakeholders engaged in global health diplomacy. By fostering international partnership and cooperation, global health diplomacy can improve the ability of both countries to tackle mental health issues together.

## WAY FORWARD

Insufficient awareness of mental health among Nepalese migrant workers worsens the condition.^15^ Several measures are needed to effectively address the mental health difficulties faced by Nepalese migrant workers. Firstly, it is vital to encourage open communication about mental health. This can be done through awareness campaigns, workshops, and support groups that are specially designed for Nepalese migrant workers. Secondly, it is important to offer accessible mental health services for Nepalese migrant workers in South Korea. This can be achieved by increasing the number of mental health professionals who are fluent in the Nepali language and knowledgeable about the cultural background of these workers. Thirdly, the South Korean government should work with non-governmental organizations and community leaders to create support networks and resources for Nepalese migrant workers. These support networks can provide a safe space for workers to express their feelings, seek advice, and access essential resources. Moreover, efforts should be made to tackle the systemic issues that cause the mental health difficulties of Nepalese migrant workers. This includes advocating for fair labor practices and adequate working conditions and addressing any forms of discrimination or prejudice faced by migrant workers. By taking these steps, South Korea can show its dedication to the well-being of Nepalese migrant workers and create a more inclusive and supportive environment for this vulnerable group.

## CONCLUSIONS

The mental health of Nepalese migrant workers in South Korea is in a critical state and requires urgent and comprehensive action. Despite their significant role in the South Korean economy, the mental well-being of Nepalese migrant workers is often neglected, resulting in lasting impacts on individuals, families, and the wider community. A complete plan consisting of strong mental health support systems, social networks, and focused awareness campaigns is vital to address the mental health disparities among this at-risk population. Global health diplomacy appears as a key instrument in solving mental health issues. Cooperation between Nepal and South Korea enabled through diplomatic channels, can lead to efficient policies and programs. Moreover, health should be a priority in the global agenda for individual well-being and social progress. Creating open communication, reachable mental health services, and support networks are essential steps in ensuring the mental well-being of Nepalese migrant workers. By tackling systemic issues and promoting international collaboration through global health diplomacy, we can create a more caring and inclusive environment for this vulnerable group, ultimately benefiting the well-being and prosperity of individuals and societies involved.
